# IRS-1 phase separation bridges IGF-1R signaling and the MITF/TYR axis to control melanogenesis

**DOI:** 10.3389/fmolb.2026.1947504

**Published:** 2026-10-29

**Authors:** Hao Di Liang, Shui Bo Xu, Yi Ting Zhou, Li Ling Zheng, Chien Yi Koay, Hooi Yeen Yap, Bee Leng Lua

**Affiliations:** 1 Department of Biochemistry and Molecular Biology, Zhejiang University School of Medicine, Hangzhou, Zhejiang, China; 2 Clarins Co. Ltd., Shanghai, China

**Keywords:** IGF-1/IGF-1R signaling, IRS-1, melanogenesis, phase separation, α-MSH/MITF axis

## Abstract

Melanogenesis is well known to be activated by the α-MSH/MITF signaling pathway. Recent studies have further revealed that the IGF-1/IGF-1R metabolic signaling axis can also induce melanin synthesis. Here, we demonstrate that IRS-1, a key downstream adaptor of both IGF-1R and the insulin receptor (IR), acts as a bridge by connecting the upstream IGF-1R signaling with the downstream MITF/TYR axis to promote melanogenesis. Importantly, this function of IRS-1 may be tissue-specific, as insulin alone fails to stimulate melanogenesis. Deletion of the self-interaction region (SAR) abolishes the phase separation of IRS-1, thereby preventing the activation of melanogenesis. Taken together, our findings reveal a novel function of IRS-1 phase separation in melanogenesis. This work not only sheds light on understanding the regulatory mechanism of melanin homeostasis but also provides a potential strategy for cosmetics companies to develop more effective whitening products.

## Introduction

Melanin homeostasis is important for normal physiological functions in organisms, such as protecting skin from UV rays and determining hair and skin color (D’Mello et al., 2016). Abnormal accumulation of melanin can lead to hyperpigmentary disorders such as melasma and ephelides (Hassan et al., 2015). Conversely, defects in melanin production results in albinism (Diallo et al., 2026). These melanin-related skin diseases often cause considerable psychological distress on patients. Therefore, elucidating the mechanisms that regulate melanin formation is of great significance for both fundamental biology and clinical therapeutics.

Melanin is synthesized by melanocytes through melanogenesis, a process modulated by multiple signaling pathways (Mucha et al., 2025). The canonical pathway is initiated by α-melanocyte-stimulating hormone (α-MSH), which binds to melanocortin-1 receptor (MC1R) and activates adenylyl cyclase, leading to increased cAMP levels and subsequent activation of protein kinase A (PKA). Activated PKA phosphorylates cAMP response element-binding protein (CREB), which translocates to the nucleus to activate transcription of microphthalmia-associated transcription factor (MITF). As the master regulator of melanogenesis, MITF upregulates expression of melanogenic enzymes—tyrosinase (TYR), tyrosinase-related protein 1 (TYRP-1), and tyrosinase-related protein 2 (TYRP-2)—to promote melanin synthesis (Hsiao and Fisher, 2014; Mucha et al., 2025).

In addition to the classical α-MSH/MC1R pathway, melanogenesis is modulated by certain metabolic and growth factor signals such as the insulin-like growth factor-1 (IGF-1)/IGF-1R axis (Hu et al., 2016). Beyond its systemic source, IGF-1 can be acquired locally by skin cells through autocrine or paracrine production, particularly from dermal fibroblasts and stratum granulosum keratinocytes (Rudman et al., 1997). Moreover, the activity of the IGF-1 pathway is enhanced under specific conditions, including aging, oxidative stress (Ko and Kim, 2019). IGF-1 promotes melanin production by elevating cAMP levels and upregulating its downstream target genes MITF and tyrosinase (Hu et al., 2016), suggesting that IGF-1/IGF-1R pathway regulates melanogenesis by activation of the cAMP/MITF axis. Notably, clinical evidence demonstrates that administration of recombinant human growth hormone (hGH) to children with short stature results in increased formation of melanocytic naevi (Pierard et al., 1996). However, the precise mechanism by which IGF-1 modulates melanogenesis remains elusive. Our studies demonstrate that the IGF-1/IGF-1R signaling pathway, but not the insulin/IR axis, specifically induces melanogenesis. In this process, insulin receptor substrate 1 (IRS-1) acts as a critical bridge that transduces signals from upstream IGF-1/IGF-1R to the downstream MITF/TYR axis, leading to activation of melanogenesis.

Phase separation is a fundamental biophysical process by which certain molecules demix from the surrounding homogeneous environment, forming two distinct phases: a dense, droplet-like phase and a dilute surrounding phase (Mangiarotti and Dimova, 2026). Molecules capable of undergoing phase separation typically contain either multiple modular interaction domains or intrinsically disordered regions (IDRs), which mediate multivalent molecular interactions and drive the formation of dense biomolecular condensates (Gao et al., 2022). Numerous membrane-less organelles such as P-bodies, Histone Locus Body as well as various signalosomes are assembled through this mechanism (Luo et al., 2018; Xu et al., 2024; Gao et al., 2022). We previously reported that phase separation of IRS-1-mediated signalosomes acts as an organizing center for insulin/IGF-1 signaling (Gao et al., 2022). However, whether IRS-1 phase separation contributes to melanogenesis is elusive. To date, no study has reported that phase separation of any endogenous signaling protein regulates melanogenesis. Using a SAR deletion mutant that fails to form IRS-1 droplets, we demonstrate that IRS-1 phase separation is essential for activation of MITF-mediated melanin synthesis. Therefore, we hypothesized that IRS-1 serves as a critical link via phase separation to connect the upstream IGF-1/IGF-1R axis to the downstream classical melanogenic MITF/TYR pathway. Our findings reveal a previously unknown role of IRS-1 phase separation in melanogenesis. This provides a new model for phase separation of an endogenous signaling protein in regulating melanogenesis. Therefore, strategies targeting this process could become a potential therapeutic approach for pigmentary disorders.

## Materials and methods

### Cell culture

Mouse B16-F10 melanoma cells used in this study was obtained from American Type Culture Collection (ATCC, CRL-6475). B16-F10 cells were cultured in Dulbecco’s Modified Eagle Medium (DMEM) (high glucose) supplemented with 10% (v/v) fetal bovine serum (FBS), 2 mM L–glutamine, 100 U/mL penicillin, and 100 mg/mL streptomycin (all obtained from Hyclone Laboratories, Logan, UT). Cells were maintained at 37 °C in a humidified atmosphere containing 5% CO_2_. Once cells reached 70%–80% confluence, cells were either subcultured or seeded according to respective experimental protocol.

### Reagents and antibodies

Melanin and α-MSH were acquired from Sigma-Aldrich (Saint Quentin Fallavier, France). The following antibodies were used for Western blotting and immunofluorescence: mouse anti-FLAG (Yeasen Biotechnology, 30503ES60), mouse anti-MITF (Abcam, ab12039), mouse anti-TYR (Santa Cruz Biotechnology, sc-20035), rabbit anti-IRS-1 (Cell Signaling Technology, 2382), rabbit anti-IGF-1Rβ (Cell Signaling Technology, 9750), mouse anti-IRβ (Santa Cruz Biotechnology, sc-57342), and mouse anti-tubulin (HuaAn Biotechnology). The fluorescent secondary antibody used was goat anti-mouse-Alexa Fluor 546 (Thermo Fisher Scientific, A-11030).

### Quantitative reverse transcriptase polymerase chain reaction (qRT-PCR)

Total ribonucleic acid (RNA) was isolated using an AFTSpin Tissue/Cell Fast RNA Extraction Kit (Abclonal, RK30120) (Cong et al., 2018; He et al., 2019). An ABScript III RT Master Mix for qPCR with gDNA Remover was used to perform reverse transcription (Abclonal, RK20429). The qPCR reactions were conducted using 2X Universal SYBR Green Fast qPCR Mix (Abclonal, RM21203) on the Roche LightCycler 480 Real-Time PCR System (RRID: SCR_018626). Each RT-qPCR experiment was technically replicated and performed with four biological replicates. All relevant primers are listed in Table 1.

**TABLE 1:** Primer sequences for qRT-PCR.

Gene	Sequence (5’-3’) - forward	Sequence (5’-3’) – reverse
*Mitf*	GAGGACTAAGTGGTCTGCGG	TGCTTTACCTGGTGCCTCTG
*Tyr*	ATCGGCCAACGATCCCATTT	TAGGTGCATTGGCTTCTGGG
*Tyrp-1*	CTGCGATGTCTGCACTGATGACTTG	TTTCTCCTGATTGGTCCACCCTCAG
*Tyrp-2*	CTTGGGGTTGCTGGCTTTTC	CGCTGAAGAGTTCCACCTGT
*Gapdh*	CATCACTGCCACCCAGAAGACTG	ATGCCAGTGAGCTTCCCGTTCAG

### siRNA transfection

Following a previously established protocol (Cong et al., 2020), B16-F10 cells at 40%-50% confluency were transfected with 100 nM siRNA using Lipofectamine RNAiMAX (Invitrogen, 13778150). The sequence of siRNAs used was: mouse IRS-1 siRNA: 5′- GCUCUUCUGCAGCUGCAUA-3’; mouse IGFR siRNA: 5′- CAGUCAUCCGUGGCUGGAAACUCUU-3’; mouse IR siRNA: 5′-CCACUGUCAUCAAUGGGCAGUUUGU-3’

### Western blot

B16-F10 cells transfected with expression plasmids were lysed in a lysis buffer (150 mM sodium chloride, 0.25 mM EDTA, 50 mM Tris, pH 7.3, 1% (w/v) sodium deoxycholate, 0.2% sodium fluoride, 1% (v/v) Triton X-100, 0.1% sodium orthovanadate) containing a protease inhibitor cocktail (Selleck Chemicals, B14001). Proteins were separated on SDS-PAGE gels and then transferred to a FluoroTrans™ polyvinylidene difluoride membrane (Pall, #BSP0106). Membranes were blocked with 5% (w/v) bovine serum albumin (BSA) for 30 min at room temperature and then hybridized with corresponding primary and secondary antibodies.

### Immunofluorescence and direct fluorescence

B16-F10 cells were seeded on coverslips in a 24-well plate and transfected with various expression constructs or siRNAs, then cultured for 18 h. After incubation, cells were fixed with 3% paraformaldehyde for 30 min. Subsequently, cell permeabilization was performed with 0.03% Triton X-100 for 30 min. Primary and secondary antibodies for immunofluorescence were diluted in final dilution buffer (5% normal goat serum, 2% FBS, 2% BSA, 1 mM CaCl_2_, 1 mM MgCl_2_ in PBS). The primary antibody incubation was performed overnight at 4 °C, followed by incubation with the secondary antibody for 2 h at room temperature. Immunofluorescence was visualized using confocal fluorescence microscopy or directly visualized for cells expressing green fluorescent protein (GFP)-tagged proteins as previously described (He et al., 2019). Images were collected at room temperature using an LSM800 Meta laser-scanning confocal microscope (Carl Zeiss, RRID: SCR_015963); 63 × 1.4 NA and appropriate laser excitations were used. The detector gain was first optimized by sampling various regions of the coverslip and then fixed for each specified channel. Once set, the detector gain value was kept constant throughout the image acquisition process. Images were analysed using Zeiss LSM Image Examiner Software.

### Melanin content determination

B16-F10 cells were plated into 24-well plates at a density of 6 × 10^4^ cells per well and incubated for 24 h. Cells were then stimulated with α-MSH (100 nM) or IGF-1 (100 ng/mL), or insulin (100 nM) in phenol-free DMEM medium containing 2% FBS for 42 h. While medium containing 2% FBS but without α-MSH served as the negative control. The pigmented medium was collected from each well, and absorbance of the medium was measured at 555 nm to measure melanin content. Concurrently, protein content was quantified using the bicinchoninic acid assay (BCA) to determine the relative melanin production. Each sample group was assayed in triplicate to ensure accuracy and reproducibility. A melanin standard curve was prepared using a 1 mg/mL melanin stock solution.

### Tyrosinase activity determination

B16-F10 cells were seeded into 12-well plates at a density of 1.5 x 10^5^ cells per well and incubated overnight. Cells were then stimulated with α-MSH (100 nM) in phenol-free DMEM medium containing 2% FBS for 42 h. While medium containing 2% FBS but without α-MSH served as the negative control. Thereafter, cells were harvested and processed according to the manufacturer’s instructions of Tyrosinase Assay Kit (Solarbio, China). Briefly, cells were lysed in tyrosinase extraction buffer and subsequently disrupted by sonication. Cell lysates were centrifuged, the resulting supernatant was mixed with the kit reagents, incubated at 37 °C for 20 min, and analyzed for absorbance at 505 nm. Tyrosinase activity was normalized to protein concentration.

### Statistical analysis

Statistical analyses were performed using GraphPad Prism 8.0.2 (GraphPad Software, Inc. RRID: SCR_002798). Results are presented as mean ± SEM or mean ± SD. Statistical significance was determined as indicated in the figure legends (*p < 0.05, **p < 0.01, ***p < 0.001, ****p < 0.0001). Data distribution was initially assessed using the Shapiro-Wilk normality test, Kolmogorov–Smirnov test and D'Agostino and Pearson omnibus normality test. Two-tailed unpaired Student’s t-test was used to compare two groups when data were normally distributed and the variances were confirmed as similar through an F test (p > 0.05). A two-tailed unpaired Student’s t-test with Welch’s correction was used when the F test indicated a significant difference in variances (p < 0.05). A Mann-Whitney U test was used when data was not normally distributed. If the variation among three or more groups was minimal, ANOVA was conducted, followed by Dunnett’s post-test or Tukey’s *post hoc* test to compare the groups.

## Results

### IGF-1 is an inducer of melanogenesis

Recent studies have revealed that IGF-1/IGF-1R signaling is involved in regulating pigmentation (Liu et al., 2020). To further elucidate the underlying mechanism, we first assessed the effects of IGF-1 and insulin on melanogenesis. B16-F10 cells, a widely used model for studying melanin synthesis, were treated with α-MSH and IGF-1 to evaluate their effects on melanin production. Since insulin was reported to inhibit melanogenesis (Benathan and Labidi, 1997), it was used as a control in addition to DMEM. Compared to insulin, which had no effect on promoting melanogenesis, both α-MSH and IGF-1 significantly increased melanin production in B16-F10 cells, although the effect of IGF-1 was less potent than that of α-MSH (Figure 1A), indicating that IGF-1 is an inducer of melanogenesis.

**FIGURE 1:**
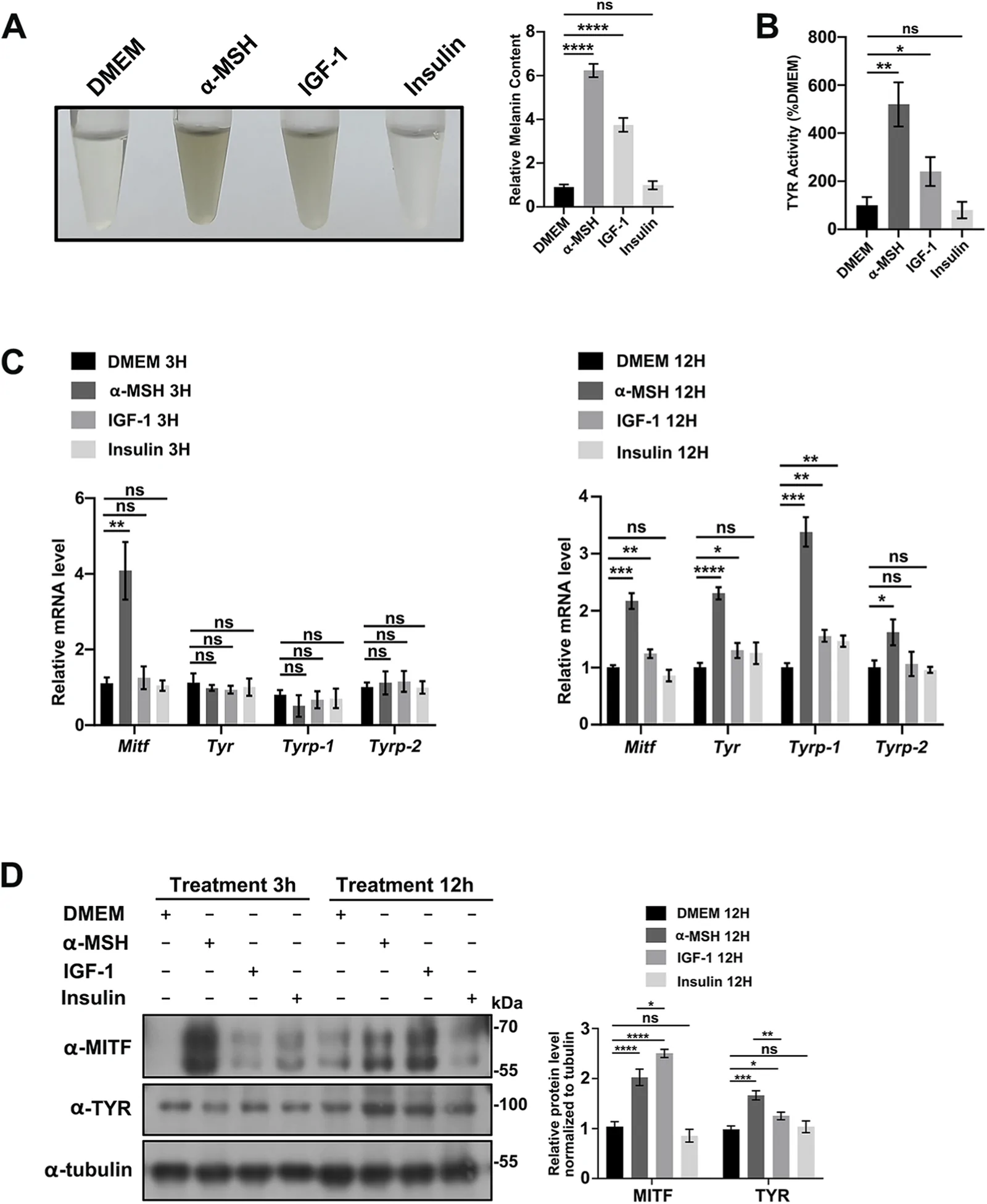
IGF-1 is an effective inducer of melanogenesis. B16-F10 cells were treated with DMEM, α-MSH, IGF-1 or insulin respectively. **(A)** Melanin contents were measured after 42 h of incubation. Left: representative image of cell suspension; right: quantification of melanin contents. n = 3. **(B)** Tyrosinase activity was measured after 42 h of incubation. Data in the bar graph represent the mean ± SD from three independent experiments. **(C)** Cells were subjected to qPCR analysis for mRNA expression of the indicated genes after 3 h (left) or 12 h (right) of incubation. n = 3. **(D)** Cells were subjected to Western blotting to analyze protein expression of indicated protein after 3 h (left) or 12 h (right) of incubation. Right: quantification of protein levels. Data represent the mean ± SD. Statistical significance was determined as indicated in the figure legends: *p < 0.05, **p < 0.01, ***p < 0.001, ****p < 0.0001.

Since melanin synthesis depends on the tyrosine metabolism pathway, tyrosinase activity serves as an indicator of pathway activation (Fan et al., 2021; King and Witkop, 1976). We found that both α-MSH and IGF-1 treatment resulted in increased tyrosinase activity, suggesting that IGF-1 and α-MSH may activate overlapping downstream melanogenic programs (Figure 1B). MITF is the key transcription factor regulating the three melanogenic enzymes, TYR, TYRP-1 and TYRP-2 (Hsiao and Fisher, 2014; Mucha et al., 2025). Therefore, we assessed the expression levels of these genes. In accordance with the enhancement of melanogenesis, both the transcription and protein levels of these genes were correspondingly upregulated (Figures 1C,D). Thus, our results confirmed that IGF-1 is indeed an important inducer of melanogenesis.

### IGF-1/IGF-1R induces melanogenesis by converging with the α-MSH/MITF signaling pathway

Having established that IGF-1 can induce melanogenesis, we next sought to test whether IGF-1/IGF-1R signaling functionally converges on the α-MSH/MITF pathway. To this end, we diminished IR and IGF-1R levels using IR- or IGF-1R-specific short interfering RNA (siRNA) oligonucleotides respectively followed by α-MSH treatment. If IGF-1/IGF-1R operates as a parallel pathway to α-MSH signaling, silencing IGF-1R should have little or no effect on melanogenesis upon α-MSH treatment, because α-MSH could bypass the IGF-1/IGF-1R axis and fully activate the downstream cascade on its own. Conversely, if IGF-1/IGF-1R converges with α-MSH signaling, downregulation of IGF-1R would attenuate melanogenesis even with α-MSH treatment, as α-MSH alone would be insufficient to fully activate the downstream cascades for melanogenesis. Knockdown of IGF-1R, but not IR, substantially reduced melanin synthesis (Figure 2A). In accordance with IGF-1-induced melanogenesis, downregulation of IGF-1R markedly reduced tyrosinase activity (Figure 2B), as well as the mRNA expression of downstream melanogenic regulators Mitf, Tyr, Tyrp-1, and Tyrp-2 (Figure 2C), and the protein expression of MITF and TYR (Figure 2D). Our results indicated that melanogenesis is specifically induced via the IGF-1R pathway rather than through non-specific activation by insulin or its receptor.

**FIGURE 2:**
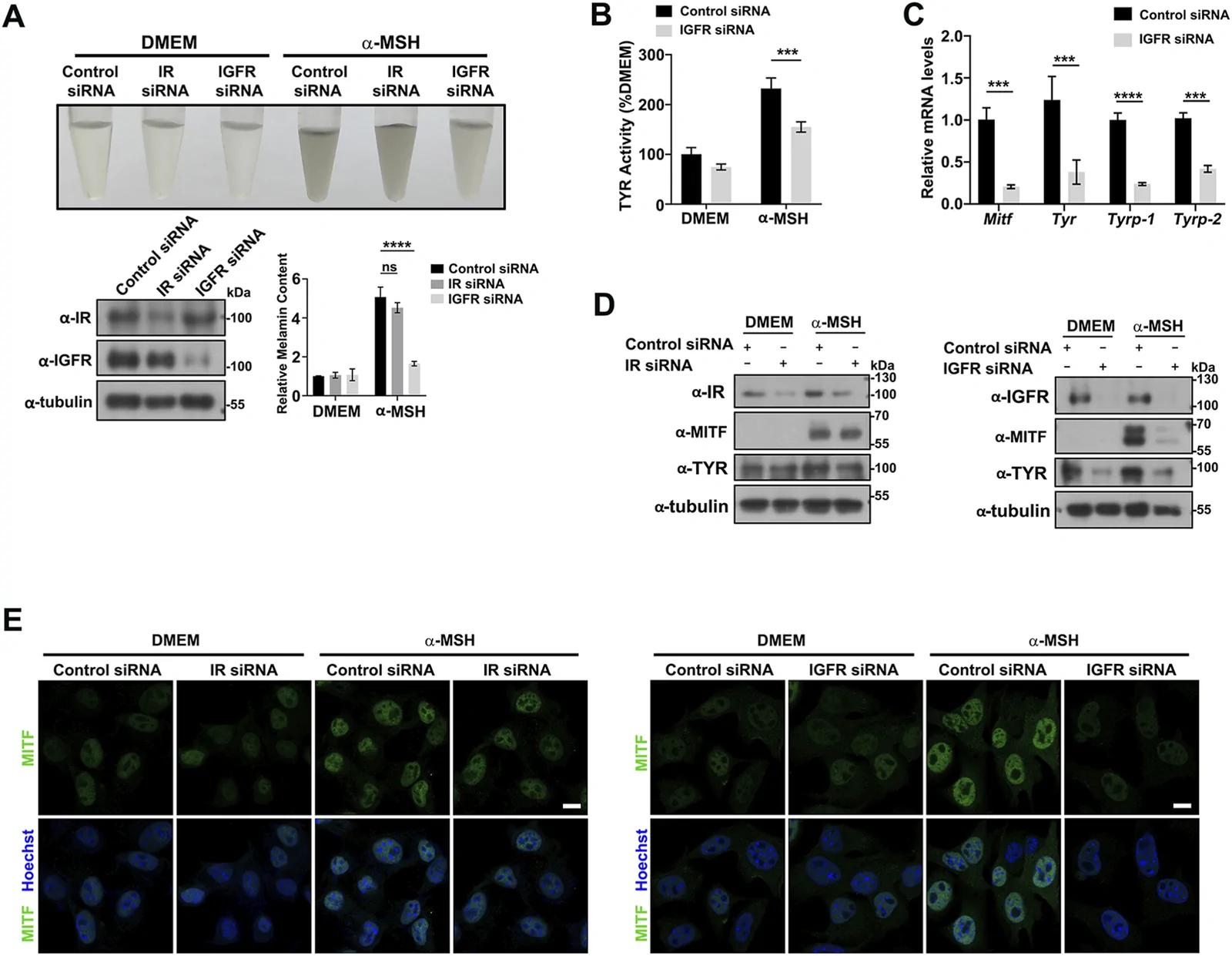
IGF-1/IGF-1R induces melanogenesis by converging with the α-MSH/MITF signaling pathway. B16-F10 cells were transfected with the indicated siRNA for 48 h followed by DMEM or α-MSH treatment. **(A)** Melanin contents were measured after 42 h of incubation. Upper panel: representative image of cell suspension; lower left: Western blot analysis of cell lysates with the indicated antibodies; lower right: quantification of melanin contents. n = 3. **(B)** Tyrosinase activity was measured after 42 h of incubation. Data in the bar graph represents the mean ± SD from three independent experiments. **(C)** Cells were subjected to qPCR analysis for mRNA expression of the indicated genes after 3 h of incubation. n = 3. **(D)** Cells were subjected to Western blotting to analyze protein expression of indicated proteins after 3 h of incubation. Left: cells transfected with IR or control siRNA; right: cells transfected with IGF-1R or control siRNA. **(E)** Immunofluorescence staining of endogenous MITF in cells after 3 h of incubation. Scale bar, 10 μm. Data represent the mean ± SD. Statistical significance was determined as indicated in the figure legends: *p < 0.05, **p < 0.01, ***p < 0.001, ****p < 0.0001.

We further confirmed this by performing immunofluorescence staining. As a transcription factor localized to the nucleus, MITF was endogenously distributed in the nuclei. Downregulation of IGF-1R, but not IR, significantly decreased MITF signaling accordingly (Figure 2E). Based on our data, we conclude that IGF-1/IGF-1R induces melanogenesis by converging with the α-MSH pathway to co-activate the downstream MITF/TYR, rather than working as an independent parallel pathway.

### IRS-1 is required for IGF-1/IGF-1R signaling to induce melanogenesis

Since IGF-1/IGF-1R signaling converges with the α-MSH/MITF pathway, we next dissected whether IRS-1, the key downstream adaptor of IGF-1R, is required for this convergence. To test this, we silenced IRS-1 followed by α-MSH treatment. If IRS-1 functions at a convergence point, its depletion should attenuate α-MSH-induced melanogenesis. We therefore downregulated the expression of IRS-1 using siRNA. Compared to the control siRNA, inhibition of IRS-1 expression significantly suppressed melanin synthesis (Figure 3A) and tyrosinase activity (Figure 3B). This reduction was accompanied by marked downregulation of downstream melanogenic gene expression, including Mitf, Tyr, Tyrp-1, and Tyrp-2 (Figure 3C), as well as decreased protein expression of MITF and TYR (Figure 3D). Consistently, immunofluorescence staining of endogenous MITF in IRS-1 downregulated cells was much reduced compared to the control cells (Figure 3E). Thus, our data reveals a novel role of the IGF-1/IGF-1R/IRS-1 signaling pathway in regulating melanogenesis, a function entirely distinct from its canonical role in metabolism.

**FIGURE 3:**
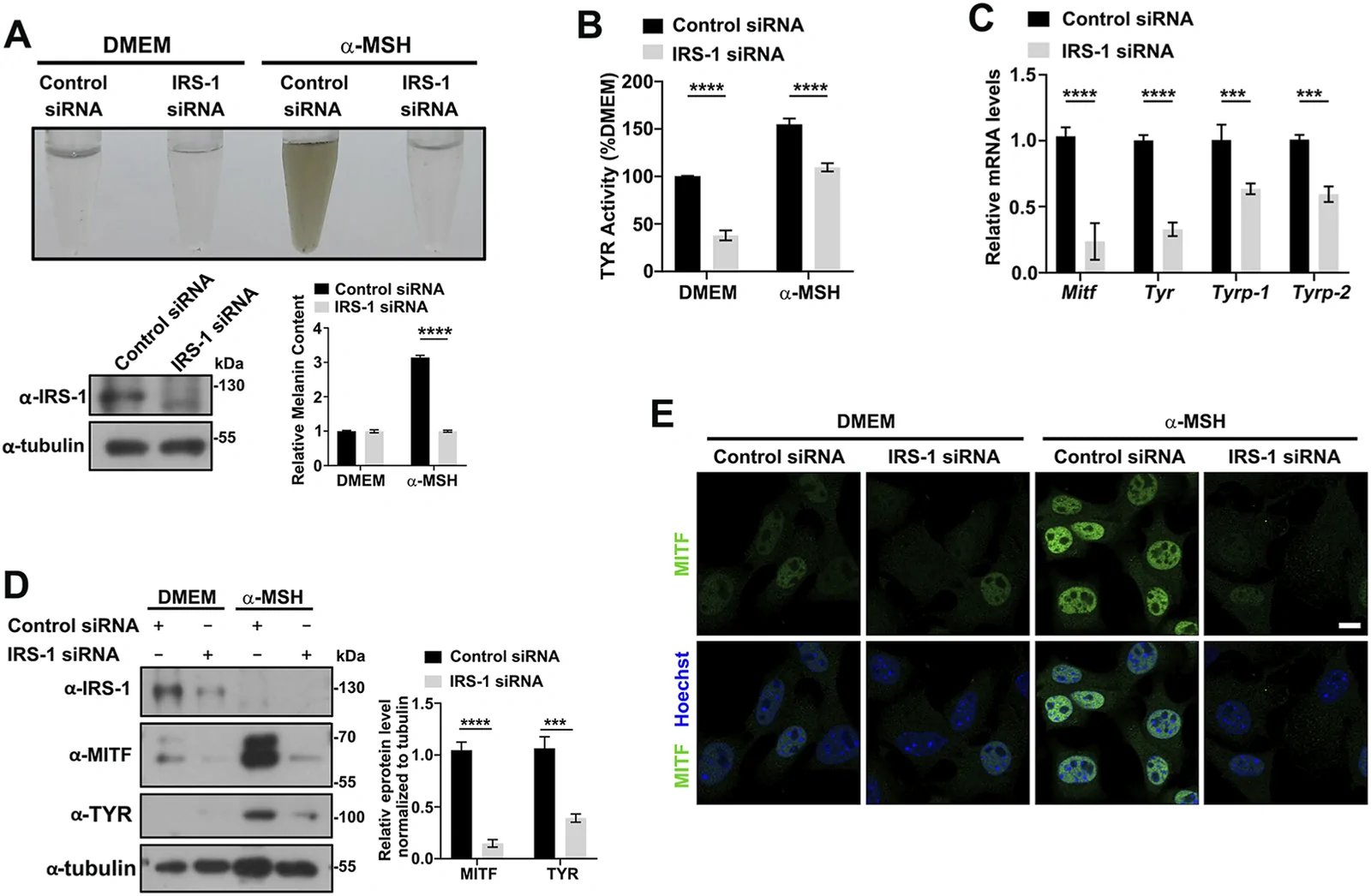
IGF-1/IGF-1R activates downstream IRS-1 to promote melanogenesis. B16-F10 cells were transfected with IRS-1 or control siRNA for 48 h followed by DMEM or α-MSH treatment. **(A)** Melanin contents were measured after 42 h of incubation. Upper panel: representative image of cell suspension; lower left: Western blot analysis of cell lysates with the indicated antibodies; lower right: quantification of melanin contents. n = 3. **(B)** Tyrosinase activity was measured after 42 h of incubation. Data in the bar graph represents the mean ± SD from three independent experiments. **(C)** Cells were subjected to qPCR analysis for mRNA expression of the indicated genes after 3 h of incubation. n = 3. **(D)** Cells were subjected to Western blotting to analyze protein levels of indicated protein after 3 h of incubation (left). Right: quantification of protein levels. n = 3. **(E)** Immunofluorescence staining of endogenous MITF in cells after 3 h of incubation. Scale bar, 10 μm. Data represent the mean ± SD. Statistical significance was determined as indicated in the figure legends: *p < 0.05, **p < 0.01, ***p < 0.001, ****p < 0.0001.

### IRS-1 bridges the upstream IGF-1/IGF-1R signaling and the downstream MITF/TYR axis to activate melanogenesis

To further validate the functional role of IRS-1 in melanogenesis, we performed ectopic expression of IRS-1 using a FLAG-tagged construct. Given that downregulation of IRS-1 inhibited melanogenesis, we hypothesized that overexpression of IRS-1 would enhance melanin synthesis. As expected, IRS-1 overexpression significantly enhanced α-MSH-induced melanin production in B16-F10 cells (Figure 4A). Consistently, tyrosinase activity was increased in IRS-1-overexpressing cells in the presence of α-MSH (Figure 4B). At the transcriptional level, IRS-1 overexpression significantly upregulated the mRNA expression of downstream regulators, namely, Mitf, Tyr, and Tyrp-2 (Figure 4C). These transcriptional changes were mirrored at the protein level, where TYR and MITF expression were increased in IRS-1-overexpressing cells (Figure 4D). This observation was further confirmed by immunofluorescence staining of endogenous MITF in GFP-IRS-1 overexpressing cells.

**FIGURE 4:**
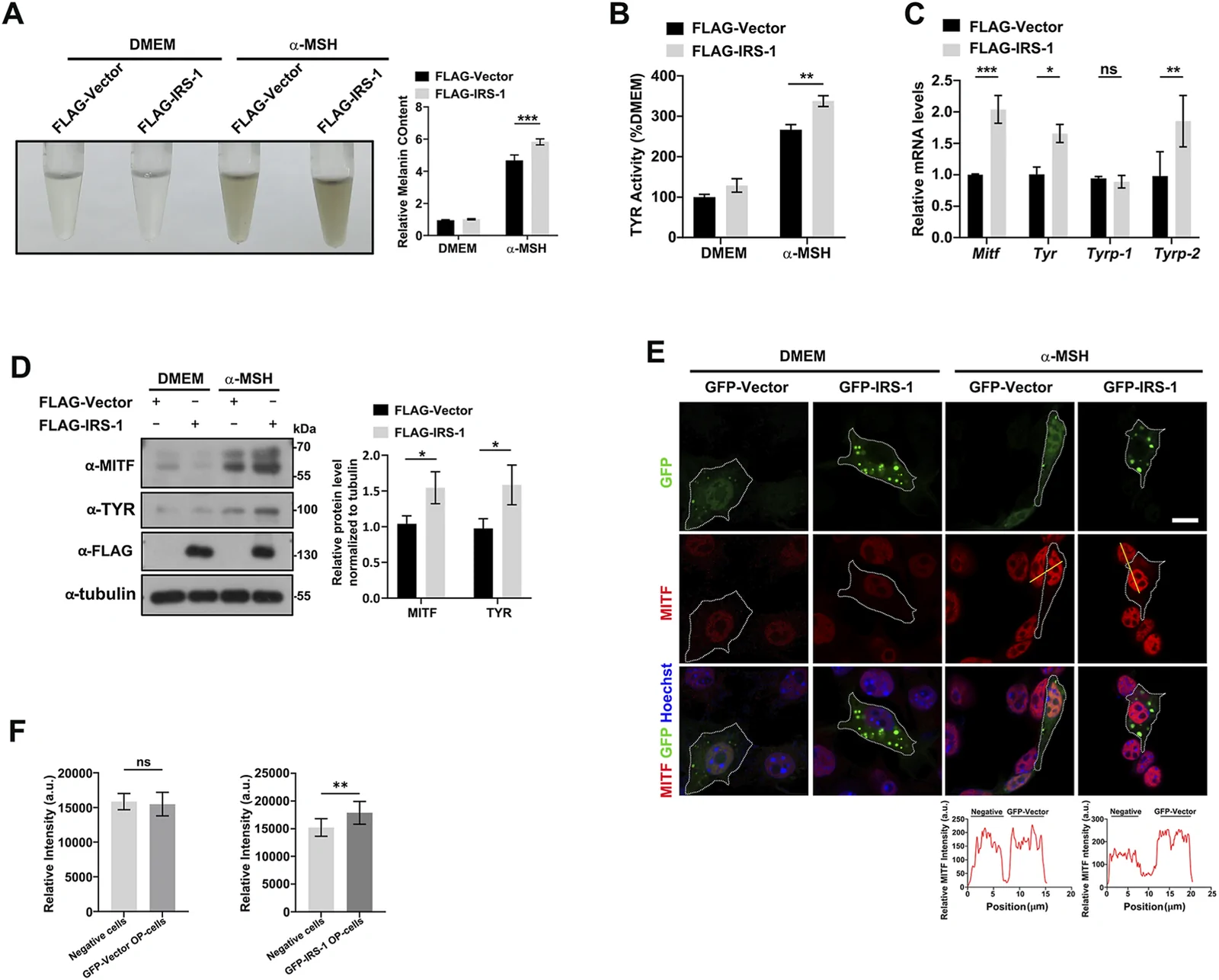
IRS-1 bridges upstream IGF-1/IGFR signaling and the downstream MITF/TYR pathway to activate melanogenesis. B16-F10 cells were transfected with FLAG-IRS-1 or control vector for 18 h followed by DMEM or α-MSH treatment. **(A)** Melanin contents were measured after 42 h of incubation. Left panel: representative image of cell suspension; right panel: quantification of melanin contents. n = 3. **(B)** Tyrosinase activity was measured after 42 h of incubation. Data in the bar graph represents the mean ± SD from three independent experiments. **(C)** Cells were subjected to qPCR analysis for mRNA expression of the indicated genes after 3 h of incubation. n = 3. **(D)** Cells were subjected to Western blotting to analyze protein expression of the indicated protein after 3 h of incubation (left). Right: quantification of protein levels. n = 3. **(E)** Immunofluorescence staining of endogenous MITF in cells after 3 h of incubation. Cells transfected with the indicated plasmid are outlined. Scale bar, 10 μm. Lower: Line scan analysis of MITF fluorescence intensity along the yellow lines across indicated plasmid-positive versus -negative cells. **(F)** Quantification of relative MITF fluorescence intensity in cells transfected with GFP-Vector or GFP-IRS-1 after 3 h of incubation. n = 11. Data represent the mean ± SD. Statistical significance was determined as indicated in the figure legends: *p < 0.05, **p < 0.01, ***p < 0.001, ****p < 0.0001.

Our previous studies have shown that ectopic expression of GFP-IRS-1 forms droplets in the cytoplasm (Gao et al., 2022). To investigate its functional impact, we examined MITF, a key transcription factor localized to the nucleus. In the absence of α-MSH stimulation, no obvious change in MITF signal was detected in GFP-IRS-1 overexpressing cells compared to GFP-IRS-1-negative cells (Figure 4E). Upon α-MSH stimulation, however, the MITF signal in GFP-IRS-1-overexpressing cells (indicated by outlines) was significantly enhanced compared to GFP-IRS-1-negative cells, where no obvious change was detected (Figure 4E). This result was further confirmed by measuring the fluorescence intensity of MITF along the yellow lines across both cell types (Figure 4F). Based on these observations, we conclude that IRS-1 serves as a critical component connecting upstream IGF-1/IGF-1R signaling to the downstream MITF/TYR pathway, thereby activating melanin synthesis.

### Phase separation of IRS-1 promotes α-MSH-induced melanogenesis

Our previous work showed that IRS-1 can undergo phase separation to form droplets, allowing it to rapidly assemble signalosomes in response to fast changes in the external environment (Gao et al., 2022). Given that IRS-1 is necessary for α-MSH-induced melanogenesis, we next investigated whether its phase separation ability is required for melanogenesis regulation. The IRS-1 protein sequence contains an IDR (intrinsically disordered region) region, which is critical for phase separation (Gao et al., 2022; Xiao and Xia, 2025). Our previous studies revealed that the 301-600 region within the IDR sequence, acting as a self-association region (SAR), is essential for the formation of IRS-1 droplets (Gao et al., 2022). Deletion of the SAR region efficiently abolishes the phase separation ability of IRS-1. Therefore, we used the SAR deleted mutant to determine whether phase separation of IRS-1 is required for melanogenesis regulation. First, we prepared the full-length wild-type (WT) and SAR deletion mutant constructs with a GFP tag (Figure 5A). Immunofluorescence staining showed that exogenously expressed IRS-1 formed spherical structures, indicating phase separation, whereas the mutant lacking the SAR region diffused throughout the cytoplasm and failed to form droplets, as we previously reported (Figure 5B).

**FIGURE 5:**
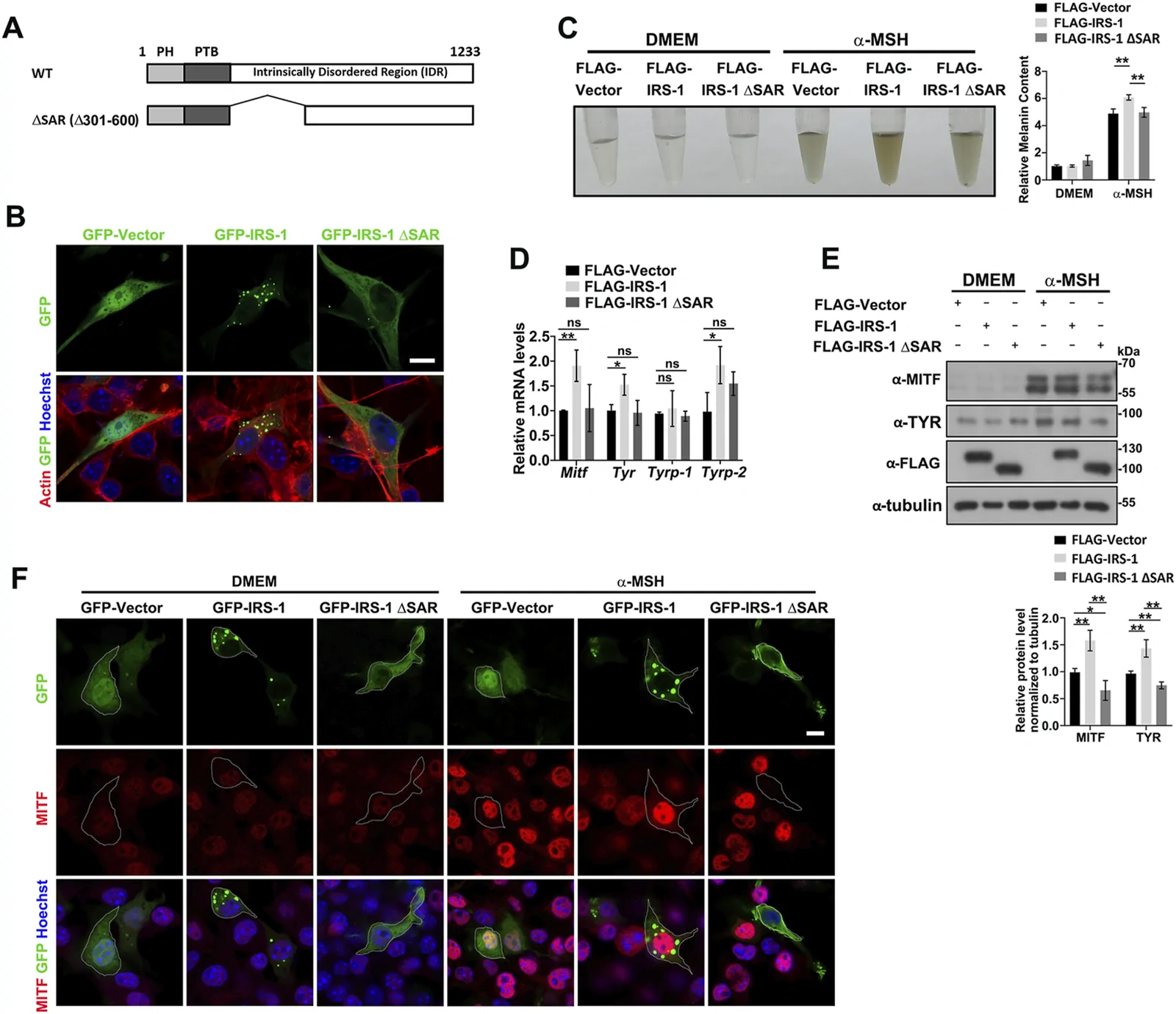
Phase separation of IRS-1 promotes α-MSH-Induced melanogenesis. B16-F10 cells were transfected with GFP-IRS-1, GFP-IRS-1ΔSAR or control vector for 18 h followed by DMEM or α-MSH treatment. **(A)** Schematic diagram of IRS-1 and its SAR deletion mutant. **(B)** Confocal images of representative cells transiently expressing the GFP-IRS-1, control GFP vector or GFP-IRS-1ΔSAR. Scale bar, 10 μm. **(C)** Melanin contents were measured after 42 h of incubation. Left panel, representative image of cell suspension; right panel: quantification of melanin contents. n = 3. **(D)** Cells were subjected to qPCR analysis for mRNA expression of the indicated genes after 3 h of incubation. n = 3. **(E)** Cells were subjected to Western blotting to analyze protein expression of the indicated protein after 3 h of incubation (upper). Lower: quantification of protein levels. n = 3. **(F)** Immunofluorescence staining of endogenous MITF in cells after 3 h of incubation. Cells transfected with indicated plasmid are outlined. Scale bar, 10 μm. Data represent the mean ± SD. Statistical significance was determined as indicated in the figure legends: *p < 0.05, **p < 0.01, ***p < 0.001, ****p < 0.0001.

Interestingly, overexpression of IRS-1 robustly increased melanin production upon α-MSH stimulation, whereas this effect was significantly reduced with overexpression of the SAR deletion mutant (Figure 5C). Consistently, the expression levels of melanogenic genes Tyr, Tyrp-1, Tyrp-2 and their upstream transcription factor Mitf (Figures 5D,E) were markedly reduced in the SAR deletion mutant compared to wild-type IRS-1. These data suggest that phase separation of IRS-1 is essential for enhancing melanogenesis. We next examined MITF expression in cells using immunofluorescence staining. Approximately 80% of the IRS-1ΔSAR overexpressing cells exhibited either undetectable or reduced MITF signaling compared to wild-type IRS-1 overexpressing cells upon α-MSH stimulation (Figure 5F). Taken together, we constructed a schematic diagram summarizing the function of IRS-1 in regulating melanogenesis. IRS-1 acts as a key node that connects upstream IGF-1/IGF-1R signaling to the downstream MITF/TYR axis through its phase separation (Figure 6).

**FIGURE 6:**
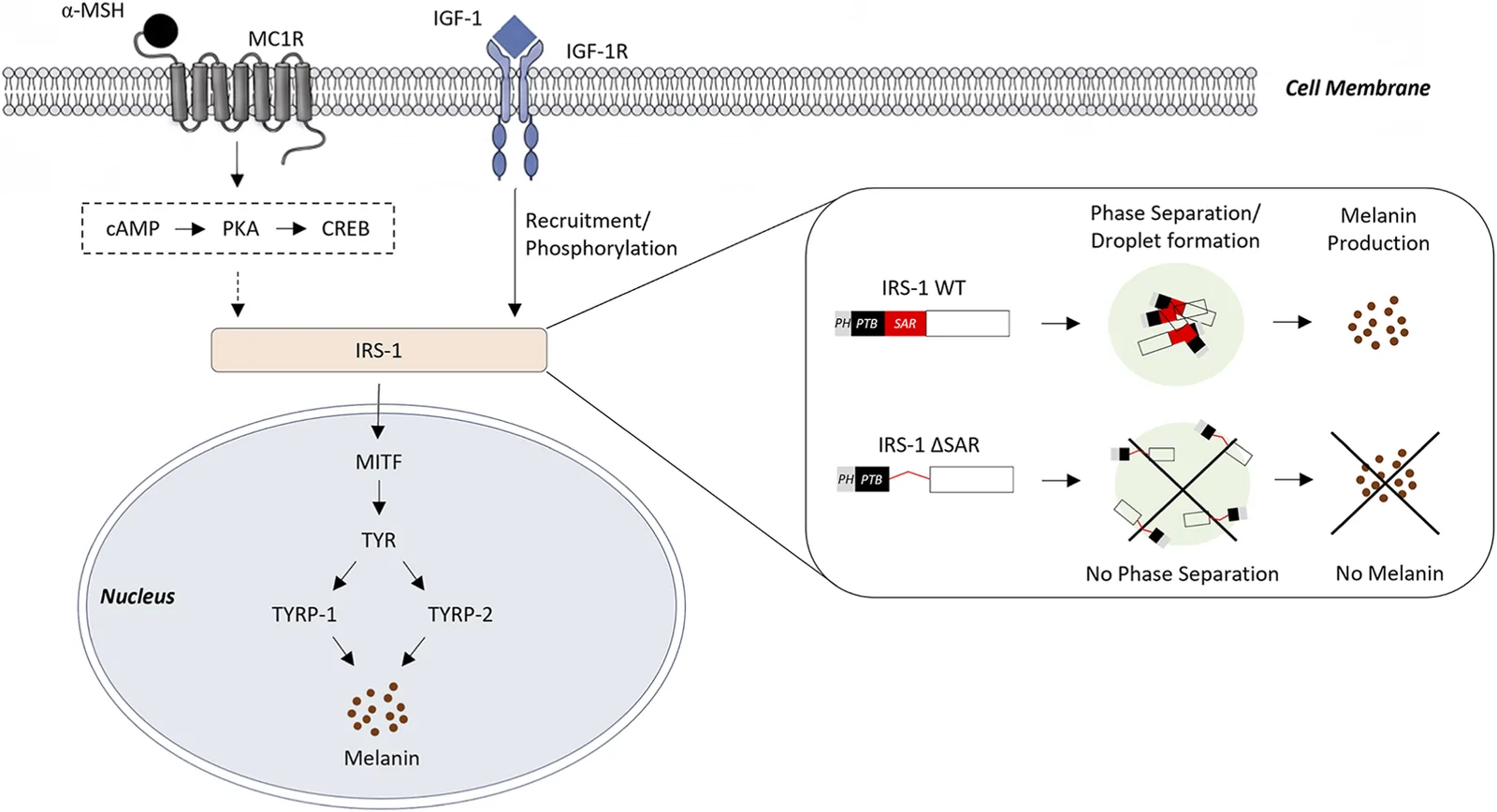
A schematic diagram illustrating the function of IRS-1 in regulating melanogenesis. IRS-1 acts as a key node connecting upstream IGF-1/IGF-1R signaling to the downstream MITF/TYR axis through phase separation. In this model, the entire cAMP/PKA/CREB module is enclosed in a dashed box, and its connection to IRS-1 is shown as a dashed arrow, indicating that whether this module lies upstream or downstream of IRS-1 remains to be determined.

## Discussion

Insulin/insulin-like growth factor (IGF) signaling modulates diverse pivotal physiological events, including differentiation, metabolism and growth (Li et al., 2021; White and Kahn, 2021). Recent studies have revealed a novel function of the IGF signaling pathway in melanogenesis (Liu et al., 2020). Here, we verify that the IGF-1/IGF-1R signaling pathway induces melanogenesis through the MITF/TYR axis. We further demonstrate that IRS-1, a downstream adaptor of IGF-1R, serves as a critical node connecting the IGF-1/IGF-1R axis to the downstream MITF/TYR signaling pathway. Importantly, we found that the IRS-1 mutant with SAR deletion is incapable of forming droplets and fails to activate melanin synthesis, indicating that phase separation of IRS-1 is essential for melanogenesis activation. These observations provide direct evidence that IRS-1 phase separation is essential for melanogenesis. Taken together, our findings reveal that phase separation of IRS-1 bridges the upstream IGF-1/IGF-1R signaling and the downstream MITF/T pathway to activate melanogenesis. To our knowledge, this is the first time to report the function of signalosome phase separation in melanin biology.

Interestingly, neither insulin treatment nor IR knockdown can stimulate α-MSH-induced melanogenesis in our study, indicating that insulin/IR signaling might be dispensable for melanogenesis. This observation is consistent with prior reports that insulin alone inhibits tyrosinase activity and fails to stimulate melanin synthesis *in vitro* (Benathan and Labidi, 1997; Fuller and Ehlers, 1984). Thus, we may have identified a tissue-specific function of the IGF-1/IGF-1R axis in melanogenesis that is not cross-coupled by the insulin/IR signaling pathway. This function is totally different from the classical regulatory crosstalk between the IGF-1/IGF-1R and insulin/IR axes in metabolism.

Insulin and IGF–1 share structural homology, and their receptors exhibit partial ligand cross-reactivity (Menting et al., 2015). Both pathways are reported to crosstalk at multiple points in regulating cell growth and metabolism. For example, at high concentration, insulin can activate IGF-1 substrates, and *vice versa* (Arnqvist, 2008). Our experiments were conducted at 100 nM insulin in melanocytes. This is a typical *in vitro* pharmacological concentration, much higher than any physiological or pathological levels *in vivo*. This supraphysiological concentration of insulin has been reported to cross-activate IGF-1R in equine lamellar tissue (Rahnama et al., 2021). However, our data demonstrate that IGF-1, but not insulin which has the potential for cross-activation at 100 nM concentration, induces melanogenesis in B16-F10 cells (Figure 1). The relationship between insulin and pigmentation is complex, since insulin resistance *in vivo* is associated with hyperpigmentation in conditions such as acanthosis nigricans (Lause et al., 2017), and pigmentation phenotypes have been reported to correlate inversely with insulin resistance in healthy individuals (Nagata et al., 2016). These seemingly contradictory findings suggest that the *in vivo* association between high insulin and hyperpigmentation is likely indirect, possibly mediated by signals released from nearby keratinocytes, rather than by direct insulin/IR signaling in melanocytes. It is possible that when insulin binds to the IGF-1 receptor in melanocytes, it does not activate the same downstream signal as when IGF-1 binds to it. This is ligand-specific signaling, a situation where the same receptor can have different effects depending on which molecule activates it.

Our finding also raises the question of why IGF-1 and insulin act so differently in melanin synthesis and what the underlying mechanism might be. Specifically, how can a given signaling pathway be deployed in a cell-type-specific manner? One possible explanation is that the receptors of these two axes have evolved tissue-specific properties, exhibiting differences in either protein expression levels or functional responses to upstream stimulation. The post-modification of IRS-1 upon IGF-1 versus insulin stimulation might be different, potentially explaining how IRS-1 selectively activates downstream melanogenic genes. Further experiments, including comparative analysis of IR and IGF-1R expression levels and their downstream substrate utilization in melanocytes, will be valuable to address this intriguing question.

We previously reported that IRS-1 forms puncta structures in cells as a result of self-interaction-mediated phase separation (Gao et al., 2023). Signalosomes driven by phase separation depend on multivalent interaction of low-complexity and disordered protein regions (Cong et al., 2026; DiRusso et al., 2022; Holehouse and Kragelund, 2024). The IRS-1 protein contains a SAR region within an intrinsically disordered region (IDR), which is critical for its self-interaction-mediated phase separation (Figure 5A). We found that wild-type IRS-1 forms droplets, whereas the SAR deletion mutant of IRS-1 is incapable of forming droplets and fails to activate melanogenesis (Figures 5B–F). Our observations clearly verify that IRS-1 serves as a critical node linking upstream IGF-1/IGF-1R signaling to the downstream MITF/TYR axis to activate melanogenesis. Therefore, we propose two key functions of IRS-1 phase separation during this process. First, it forms droplets by concentrating key signaling components to integrate the signal; Second, it can quickly respond to external stimuli by assembling or disassembling droplets. Thus IRS-1 phase separation allows melanocytes to rapidly respond to IGF-1 stimulation and transduce signals to downstream melanogenic target genes. PI3K/AKT and Ras-MAPK are two main downstream pathways of IRS-1. Activation of the p38/JNK MAPK pathway has been shown to promote melanogenesis (Wusiman et al., 2025), whereas the role of the PI3K/AKT remains controversial, with reports of both positive (Madi et al., 2013) and negative regulation (Lee et al., 2012). Further investigation will shed light on how these pathways contribute to IRS-1-mediated melanogenesis.

This novel function of IRS-1 phase separation in melanogenesis not only provides a new facet of pigmentation regulation but also suggests potential translational applications for developing skin-whitening agents by inhibiting IRS-1 phase separation. Conversely, drugs designed to promote IRS-1 phase separation might represent a novel therapeutic strategy for patients with defects in melanin production, such as those with albinism. Further studies should aim to investigate the therapeutic potential of targeting IRS-1 phase separation in pigmentary disorders.

## Data Availability

The raw data supporting the conclusions of this article will be made available by the authors, without undue reservation.
